# Discrepancy between Measured Serum Total Carbon Dioxide Content and Bicarbonate Concentration Calculated from Arterial Blood Gases

**DOI:** 10.7759/cureus.398

**Published:** 2015-12-07

**Authors:** Youngho Kim, Larry Massie, Glen H Murata, Antonios H Tzamaloukas

**Affiliations:** 1 Medicine Service, Sandoval County Regional Medical Center, Rio Rancho, New Mexico; 2 Department of Medicine, University of New Mexico School of Medicine; 3 Pathology Service, Raymond G. Murphy VA Medical Center, Albuquerque, New Mexico; 4 Raymond G. Murphy VA Medical Center, Albuquerque, New Mexico; 5 University of New Mexico School of Medicine

**Keywords:** total co2 concentration, bicarbonate concentration, carbonic acid/bicarbonate pk', acid-base status, acidemia, hypercapnia, respiratory acidosis, metabolic acidosis

## Abstract

Large differences between the concentrations of serum total carbon dioxide (TCO_2_) and blood gas bicarbonate (HCO_3_^-^) were observed in two consecutive simultaneously drawn sets of samples of serum and arterial blood gases in a patient who presented with severe carbon dioxide retention and profound acidemia. These differences could not be explained by the effect of the high partial pressure of carbon dioxide on TCO_2_, by variations in the dissociation constant of the carbonic acid/bicarbonate system or by faults caused by the algorithms of the blood gas apparatus that calculate HCO_3_^-^. A recalculation using the Henderson-Hasselbach equation revealed arterial blood gas HCO_3_^-^ values close to the corresponding serum TCO_2 _values and clarified the diagnosis of the acid-base disorder, which had been placed in doubt by the large differences between the reported TCO_2_ and HCO_3_^-^ values. Human error in the calculation of HCO_3_^-^ was identified as the source of these differences. Recalculation of blood gas HCO_3_^-^ should be the first step in identifying the source of large differences between serum TCO_2_ and blood gas HCO_3_^-^.

## Introduction

The measured total carbon dioxide (TCO_2_) in serum has three main components, bicarbonate anion (HCO_3_^-^), dissolved carbon dioxide (dCO_2_), and carbonic acid (H_2_CO_3_), which is the hydrated form of carbon dioxide. The concentration of dCO_2_ is calculated from the partial pressure of CO_2_ (pCO_2_). Not taking into account the concentration of H_2_CO_3_ in this calculation is the source of a negligible error. In body fluids, one H_2_CO_3_ molecule is at equilibrium with 340 molecules of dCO_2_ under normal temperature and physiologic conditions [1]. At a pH of 7.40, the concentration of HCO_3_^-^ is 20-fold higher than the concentration of dCO_2_ and the concentration of H_2_CO_3_ is 1.2/340 or 0.004 mmol/L when the TCO_2_ concentration is 25.2 mmol/L and the HCO_3_^-^ level is 24 mmol/L. The ratio HCO_3_^-^/dCO_2_ decreases progressively as the pH decreases but remains routinely high. Consequently, the concentrations of TCO_2_ in a serum sample and of HCO_3_^-^ in a simultaneously drawn blood gas sample should differ only slightly, with TCO_2_ typically exceeding HCO_3_^- ^by less than 3 mmol/L. 

In a sample of blood gases, the concentration of dCO_2_ is expressed by multiplying the pCO_2_ by a proportionality coefficient *S* converting units of partial pressure to units of molar concentration. The value of HCO_3_^-^ in blood gases is routinely obtained by entering the measured values of pH and pCO_2_ in an algorithm representing the Henderson-Hasselbach equation, the general expression of which is as follows:

                          pH = pK’ + log(HCO_3_^-^/[*S*xpCO_2_])                      {1}

where pK’ is the negative logarithm of the first dissociation constant of H_2_CO_3_. The general expression of HCO_3_^-^ concentration is obtained by rearranging equation 1 as follows:

                        HCO_3_^-^ = *S*xpCO_2_x10^(pH - pK)^                               {2}

The coefficient *S* is not constant. The temperature and composition of the solution tested are factors affecting this coefficient. At body temperature, the coefficient *S *obtains the value 0.0301 mmol/L per mm Hg in blood [1]. The pK’ of H_2_CO_3_ in aqueous solutions is around 3.5 [2]. The calculation of this pK’ in biological fluids assumes that H_2_CO_3_ consists of the whole dCO_2_. Therefore, the apparent value of pK’ is at or very close to 6.1 under normal conditions [1].

Large differences between simultaneously obtained concentrations of serum TCO_2_ and arterial blood HCO_3_^-^ are encountered in certain instances. These differences complicate the evaluation of the acid-base status of patients and may lead to diagnostic and therapeutic errors. One potential source of the discrepancy is rooted in the erroneous assumption that the pK’ of H_2_CO_3_ is constant at 6.1. It was suggested that this pK’ is the cause of erroneous calculation of HCO_3_^- ^in blood gases [3].

Differences in the concentrations of closely obtained TCO_2 _and HCO_3_^- ^may have other origins in addition to a wrong value of pK’ applied in the calculation of HCO_3_^-^. A systematic search for the cause of these differences is merited. We report a patient presenting with a large difference between serum TCO_2_ and arterial blood gas HCO_3_^-^ in two consecutive sets of serum and blood gas samples. By our calculations, the pK’ was not the cause of this difference. A different reason for the difference was detected and led to the correct diagnosis of the acid-base disturbance, which was in doubt because of the TCO_2_/HCO_3_^-^ discrepancy.

## Case presentation

### Patient

Permission to report this case was obtained from the Raymond G Murphy VA Medical Center Institutional Human Research Committee. This paper was approved as a case report with waiving of the informed consent with the proviso that all identifying information was removed from the text.

A 61-year-old man with acute confusion and shallow and infrequent respirations was transferred to this hospital from a nursing home. He carried the diagnosis of alcoholic cirrhosis with ascites. One year prior to this admission, he had a small bowel resection with ileostomy. Six months later, he had surgical repair of an incarcerated inguinal hernia. 

In the days prior to admission, he had consumed an unknown number of oxycodone tablets. On admission, his temperature was 36.9° C, blood pressure - 132/68 mm Hg, and pulse rate - 89 per minute. Urine toxicology revealed large concentrations of opioids. Acute respiratory failure was diagnosed. He underwent tracheal intubation and was ventilated. Table 1 shows arterial blood acid-base parameters and serum TCO_2_ in the first two sets of simultaneously drawn samples of arterial blood gases and serum. Arterial blood gas values were determined in a satellite “point-of-care” instrument almost immediately after collection of the samples. The concentration of serum TCO_2_ exceeded that of arterial blood gas HCO_3_^-^ by 10.7 mmol/L in the first set of blood samples and by 7.6 mmol/L in the second set. In the first and second serum samples, respectively, sodium concentrations were 139 and 138 mmol/L, chloride concentrations were 102 and 103 mmol/L, and anion gaps were 8 and 9 mEq/L. Lactate level was 1.1 mmol/L in the first blood sample. 

**Table 1:** Reported simultaneously obtained serum and arterial blood gas acid-base parameters

Study	Arterial pH	Arterial pCO_2_ mm Hg	Arterial HCO_3_^-^ mmol/	Serum TCO_2_ mmol/L
1st	6.91	149	18.3	29.0
2nd	7.11	74	18.4	26.0

The patient’s respiratory acidosis improved rapidly with ventilation. He was extubated the next day. His mental status improved slowly and returned to baseline in four days. The concentration of serum TCO_2_ was between 24 and 27 mmol/L in samples obtained after the first two samples during this hospitalization.

### Investigations of the source of the discrepancy between TCO2 and HCO3-

Identification of the source of the difference between serum TCO_2_ and arterial blood HCO_3_^-^ was attempted in three successive steps:

The Potential Effect of Elevated pCO_2_

**Figure 1:**
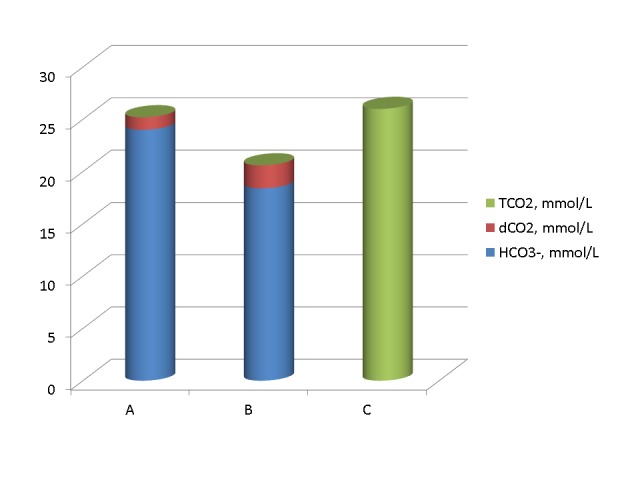
Components of total carbon dioxide content A: Idealized normal subject. B: Arterial blood gas dCO_2_, plus H_2_CO_3_, plus HCO_3_^- ^in the first set of blood tests in the patient presented in this report. C: Serum TCO_2_ in the first set of blood tests in the patient of this report. The concentration of arterial dCO_2_ was substantially higher in B than in A. Despite adjustment for the high dCO_2_, the measured concentration of TCO_2_ in serum (C) remained substantially higher than its computed concentration in the blood gasses (B).

The concentration of TCO_2_ is considered at first approximation to be equal to the sum of the concentrations of dCO_2_ and HCO_3_^-^. Because it is a linear function of pCO_2_, dCO_2_ accounts for progressively larger differences between serum TCO_2_ and HCO_3_^-^ at progressive levels of hypercapnia. We tested whether dCO_2_, calculated as 0.0301XpCO_2_, accounted for the differences between serum TCO_2_ and blood gas HCO_3_^-^ in our patient. Figure 1 shows this comparison for the first set of measurements. The figure shows the sum of blood gas HCO_3_^-^, dCO_2_, and H_2_CO_3_ in an idealized normal subject with an HCO_3_^-^ of 24 mmol/L and a dCO_2_ of 1.2 mmol/L and in the first set of arterial blood gases in the patient of this report, plus the measured TCO_2_ in the first blood sample of this patient.

**Figure 2:**
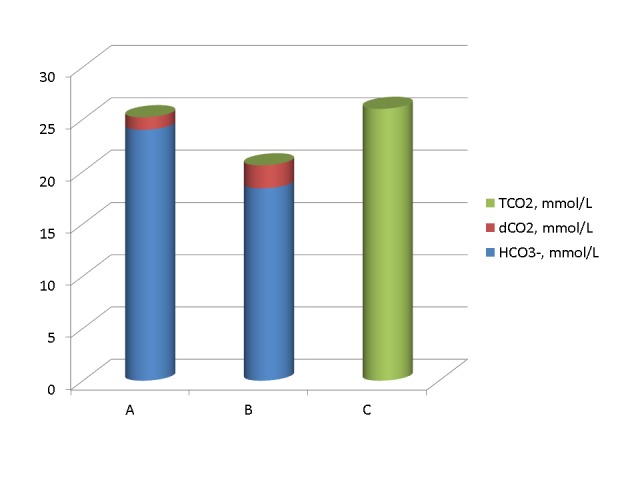
Components of total Carbon dioxide content A. Idealized normal subjects. B. Arterial blood gas HCO_3_^-^, plus dCO_2_, plus H_2_CO_3_ in the second set of blood tests in the patient of this report. C. Measured serum TCO_2_ in the second set of blood tests in the patient of this report. The measured serum TCO_2_ (C) remained substantially higher than the calculated blood gas TCO_2 _(B).

Figure 2 shows the comparison between the sum of blood gas HCO_3_^-^ , dCO_2_, and H_2_CO_3_, plus the measured serum TCO_2_ in the second set of measurements and in the idealized normal subject.

Figures 1 and 2 have two noticeable findings. The first finding is that the concentration of H_2_CO_3_ was truly negligible. The concentration of H_2_CO_3_ should be shown in red. Red colour columns did not appear in the figures even at a pCO_2_ of 149 mm Hg. The main finding of Figures 1 and 2 is that accounting for dCO_2_ reduced the difference of the concentrations between serum TCO_2_ and arterial blood HCO_3_^-^ but did not eliminate it. Substantial parts of this difference remained unaccounted for. 

Potential Effect of Changing pK’

**Figure 3:**
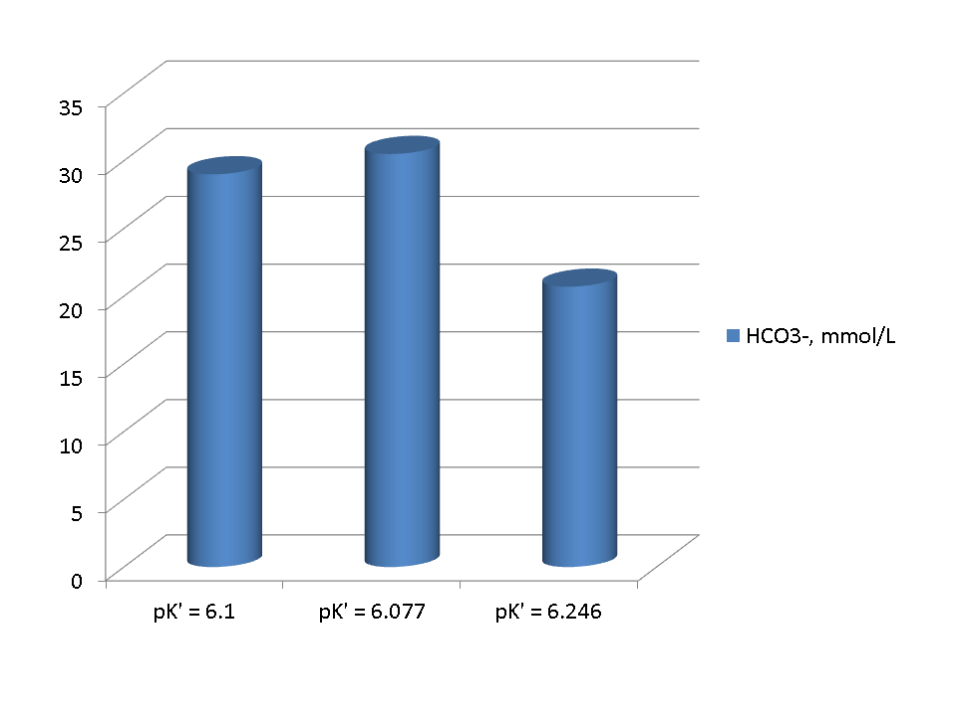
Bicarbonate values calculated at a pH of 6.91 from a pCO2 of 149 mm Hg by various pK' values High pK' values, observed at low temperatures and low pH values result in low values of calculated HCO_3_^-^.

The pK’ of H_2_CO_3_ in various solutions is not a fixed value. Variables affecting this pK’ include the temperature, acidity, and ionic strength of the solution tested [3-7]. The first two variables have been studied extensively [3-6]. The pK’ value increases with increasing acidity and decreasing temperature. Increasing the pK’ value in equation 2 lowers the value of HCO_3_^-^ calculated from it for the same value of pCO_2_. In the chapter by Madias and Cohen [1], Table 1-3 shows apparent pK’ values in human plasma at temperatures between 10 and 40° C and pH values between 7.0 and 7.6. In this table, the lowest plasma pK’, at a pH of 7.60 and a temperature of 40° C, was 6.077 while the highest pK’, at a pH of 7.00 and a temperature of 10 °C, was 6.246. Figure 3 shows the values of HCO_3_^-^ calculated from equation 2 for a pCO_2_ of 149 mm Hg and pK’ values of 6.1, 6.077, and 6.246. The figure shows substantial differences between these values. The lowest value of HCO_3_^-^ was calculated for a pK’ of 6.246 and the highest value for a pK’ of 6.077.

The purpose of Figure 3 was to show the direction of the changes in the HCO_3_^-^ concentration that result exclusively from changes in the pK’. Consequently, the pH was kept at 6.91 and the temperature was kept at 37° C in all three calculations. Correction for the effect of temperature on the pCO_2_ and use of the correct pK’ for each temperature and pH in equation 2 would change the differences shown in this figure and could potentially increase them. Therefore, a variation in the pK’ can potentially account for large variations in HCO_3_^-^ and large differences between serum TCO_2_ and blood gas HCO_3_^-^. 

We tested the effect of changing pK’ on the differences between serum TCO_2_ and arterial blood gas HCO_3_^- ^in the patient presented in this report in two sets of calculations. In the first set, we repeated the calculations of HCO_3_^-^ using a pK’ of 6.1 by the following equation:

                HCO_3_^-^ = 0.0301xpCO_2_x10^(pH – 6.1)^                                 {3} 

**Figure 4:**
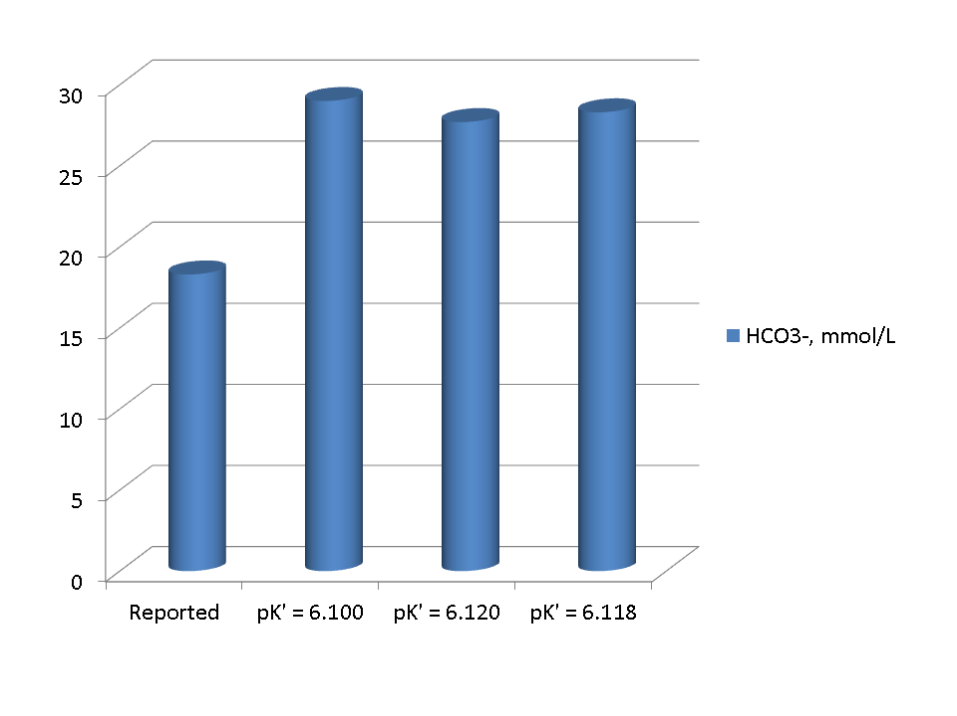
Bicarbonate concentrations in the first arterial blood sample reported and calculated by the use of three different pK' values The three calculated HCO_3_^-^ values were substantially higher than the reported value and very close to each other and to the measured serum TCO_2_.

In the second set of calculations, we calculated the approximate pK’ value at a pH of 6.91 by performing a linear regression on pH and pK’ values at a temperature of 37° C in Table 1-3 of the chapter by Madias and Cohen [1]. We obtained the following regression: pK’ = 6.410 – 0.042xpH, r = -0.994. The pK’ values calculated by this regression are 6.120 for a pH of 6.91 and 6.114 for a pH of 7.11. Figure 4 shows HCO_3_^-^ values reported and calculated from equations 3 and 2 for pK’ values of 6.1 and 6.120 in the first set of measurements. The calculated values were substantially higher than the reported HCO_3_^-^ value and very close to each other and the measured serum TCO_2_ of 29 mmol/L.

**Figure 5:**
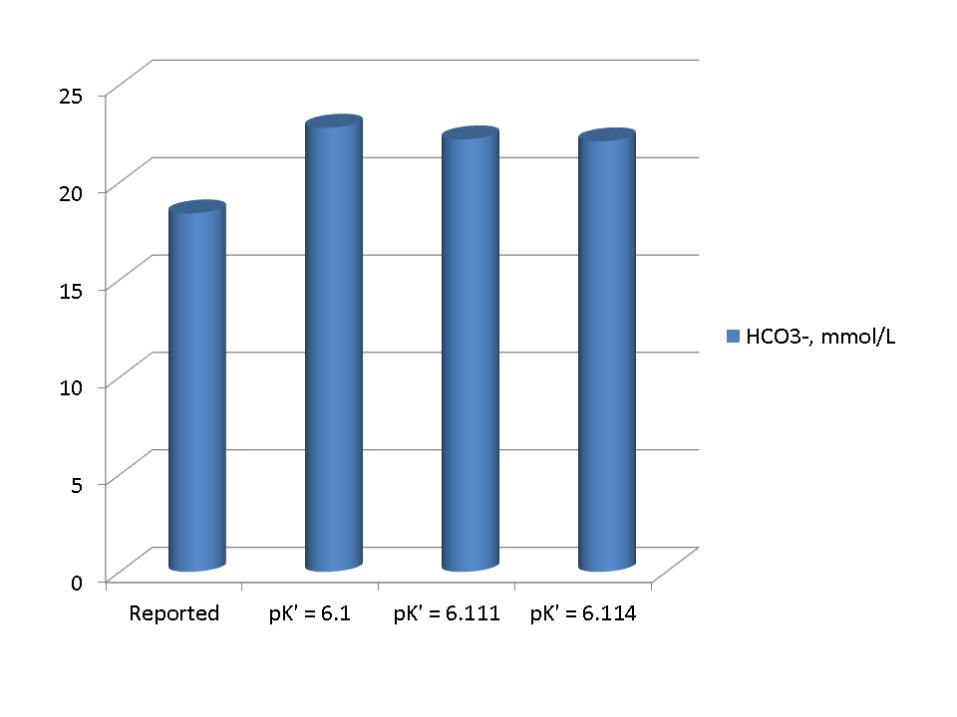
Bicarbonate concentrations in the second set of arterial blood gasses reported and calculated by three different pK'. In the first and second set of blood gasses alike, the calculated HCO_3_^-^ values were substantially higher than the reported value and very close to each other and to the corresponding measured serum TCO_2_.

Figure 5 shows HCO_3_^- ^values reported and calculated from equations 3 and 2 for pK’ values of 6.1 and 6.114 in the second set of measurements. Again, the calculated values were substantially higher than the reported HCO_3_^-^ value and very close to each other and the measured serum TCO_2_ of 26 mmol/L. 

The pK’ value that is required to obtain an HCO_3_^-^ of 18.3 mmol/L, calculated from the Henderson-Hasselbach equation for a pCO_2_ of 149 mm Hg and a pH of 6.91 is 6.299, a value substantially higher than any of the values provided in the table published by Madias and Cohen [1].  From equation 1, the pH value required for the calculation of an HCO_3_^-^ of 18.3 mmol/L at a pCO_2_ of 149 mm Hg is 6.71 when the pK is 6.1. We concluded that it is unlikely that the observed difference between serum TCO_2_ and arterial blood HCO_3_^-^ was due to variation in the value of pK’.  

Potential Effect of Instrument Calibration Error or Human Error

From the previous calculations, we concluded that the reported blood gas HCO_3_^-^ values were in error. The similarity of the calculated blood gas HCO_3_^-^ values in the first and second sets of measurements suggested a possibility that the error may be in the algorithms of the blood gas apparatus. We investigated whether the error in the calculations of HCO_3_^-^ was due to issues related to the blood gas apparatus or to errors of the operators of the blood gas apparatus by repeating the calculations using the logarithms for the pK' and HCO_3_^-^ calculations shown in the manual of the apparatus. At a temperature of 37° C, the pK’ values calculated by these algorithms are 6.118 for a pH of 6.91 and 6.114 for a pH of 7.11. Figures 4 and 5 show the calculated blood gas HCO_3_^-^ values in the first and second set of measurements by the use of these pK’ values. These values are also very close to the other calculated values and the values of the reported serum TCO_2_. We concluded that the error in the reported HCO_3_^-^ values cannot be attributed to the algorithms of the blood gas apparatus.

## Discussion

Acid-base disorders indicate health challenges that must be addressed because they result from profound respiratory or metabolic derangements and can be life-threatening. The first step in the management of an acid-base disorder is a correct diagnosis based on accurate measurement or calculation of the acid-base determinants in the blood and on the proper interpretation of these determinants [8]. The diagnosis is based on the observed combination of the measured value of blood pH and the measured or computed values of the pivotal carbonic acid/bicarbonate buffer system [9]. The patient presented in this report illustrates the difficulty encountered when measured and computed acid-base parameters lead to different diagnostic acid-base categories. A pathway leading to deciphering this difficulty is detailed.

In the first two sets of serum and arterial blood gas measurements of our patient, substantial differences were found between the reported concentrations of serum TCO_2_ and arterial blood HCO_3_^-^ (Table 1). These differences created differing impressions of the underlying acid-base disorder. The arterial blood gases suggested a picture of mixed respiratory and metabolic acidosis while the combination of arterial pH and pCO_2_, plus serum TCO_2_, indicated acute respiratory acidosis alone [10]. Previous medical history assists in the diagnosis of acid-base disorders [9]. In our patient, previous extensive gut surgery could have been the cause of metabolic acidosis, but his mental status on admission did not allow questioning about recent bowel movements. In any event, the critical issue in this and all other cases with similar findings is an identification of the correct one among the two conflicting acid-base parameters. Variations in the pK’ of the carbonic acid/bicarbonate system have been extensively studied as a source of TCO_2_/HCO_3_^-^ differences.

Variation of pK’ with temperature and acidity of the biological fluid tested was documented in both humans [3-6] and animals [11-12]. It was reported that variation in the pK’ values results in substantial variation of the HCO_3_^-^ values calculated by the Henderson-Hasselbach equation [13]. Observed differences between serum TCO_2_ and HCO_3_^-^ values calculated by using the Henderson-Hasselbach equation with the pK’ fixed at 6.1 were attributed to errors in the value of HCO_3_^-^ secondary to varying pK’ [14-20]. This approach suggests that the correct acid-base value for diagnosing the acid-base disorder and assessing its magnitude is the serum TCO_2_, the measurement of which is not affected by changes in pK’. 

The concept that the bicarbonate/carbonic acid pK’ is the main cause of discrepancies between serum TCO_2_ and blood gas HCO_3_^- ^has been challenged [21]. One criticism of this concept is that the scale of the pK’ change that has been calculated for changes in blood acidity and temperature is not large enough to explain large differences between TCO_2_ and HCO_3_^-^ [22]. As noted, higher values of pK’ resulting from high acidity or low temperature of the solution tested lead to lower values of HCO_3_^-^ calculated by the Henderson-Hasselbach equation. Studies in critically ill patients found only a small variation of the pK’ around 6.1 [23-26]. These studies suggest that varying pK’ is not the cause of large differences between TCO_2_ and HCO_3_^-^. 

Another potential source of differences between serum TCO_2_ and blood gas HCO_3_^-^ is an analytical overestimation of TCO_2_ [27]. TCO_2_ is measured by converting at a temperature of 37° C essentially all CO_2_ in the tested sample to HCO_3_^-^ by a complex enzymatic method producing NAD^+^ from a known substrate of NADH under the influence of HCO_3_^-^. The remaining NADH is measured by reflectance spectrophotometry [28]. A large list of tested medications and organic compounds showed no interference with this assay [29]. However, overestimation of TCO_2_ was reported in some cases and was attributed to interference by organic acids or carbamino compounds [30-31]. Based on this interpretation of the source of differences between serum TCO_2_ and blood gas HCO_3_^-^, Halperin and coauthors proposed that these differences are not caused by variations in the pK’ and that the level of HCO_3_^-^, rather than that of TCO_2_, is the correct measurement [32]. In a recent case report, serum TCO_2_ was consistently low in a patient with normal blood pH and HCO_3_^-^ [33]. The presence in the patient’s serum of paraproteins causing turbidity and interfering negatively with the enzymatic measurement of TCO_2_ was identified as the source of the erroneously low TCO_2_ values in this report. 

We evaluated the pK’ as the potential source of the difference between serum TCO_2_ and blood gas HCO_3_^-^ in the patient presented in this report. The arterial blood pH in the first set of measurements, at 6.91, was lower than the lowest pH value in Table 1-3 of the Madias and Cohen chapter [1]. We calculated the pK’ value for a pH of 6.91 by performing a linear regression of pH on the pK’ values reported in the same table at a temperature of 37 °C. An apparent linearity of the relationship pK’/pH within “physiologic” pH values has been reported [34]. Changing ionic strength is the source of a curvilinear relationship pK’/pH [7]. However, the ionic strength of the blood of our patient was within normal limits. The slope of the pK’/pH regression that we performed was, at -0.042, within the range of slopes (-0.05 to -0.04) reported in the literature [17]. The pK’ values that we calculated were almost identical to the values calculated by the algorithms of the blood gas apparatus. HCO_3_^-^ values calculated using a pK’ of 6.1 and pK’ values calculated from our pH/pK’ regression analysis of the Madias and Cohen Table 1-3 and from the algorithms of the blood gas apparatus were very close to each other and to the corresponding serum TCO_2_ values clarifying the diagnosis of the acid-base disorder and identifying human error as the source of the observed TCO_2_/HCO_3_^-^ differences. 

Based on the closeness of the HCO_3_^-^ values calculated by using a pK’ of 6.1 and pK’ values obtained by other methods, we propose that the first step in identifying the source of large TCO_2_/HCO_3_^-^ differences should be a recalculation of the blood gas HCO_3_^-^ value using equation 3 with a pK’ of 6.1. This calculation is simple and will provide an easy answer to the question of error. If large differences TCO_2_/HCO_3_^-^ persist after the first recalculation of the blood gas HCO_3_^-^, the second step, only in severe hypercapnia, should be to calculate the dCO_2_ as 0.0301xPCO_2_. Persistence of large TCO_2_/HCO_3_^-^ differences should lead to a new calculation of the HCO_3_^-^ using the algorithms of the blood gas apparatus because they may still reveal substantial differences in extreme acid-base disturbances. Persisting differences, unexplained by changes in pK’ or pCO_2_, require a systematic investigation of the timing of blood gas and serum samples in patients with unstable acid-base status and investigation of the handling of the blood samples, with emphasis on the blood gas sample (temperature, time period between drawing and measuring the sample, etc). If sampling issues are discovered, a new simultaneous set of blood gases and serum samples should be obtained. If differences persist in this new set, conditions affecting the measurement of TCO_2_ should be investigated [30-31, 33].

## Conclusions

Neither a variation in the apparent carbonic acid pK’, nor a high PCO_2_ value appears to provide adequate explanations for very large differences between measured serum TCO_2_ and calculated blood gas HCO_3_^-^. Although analytical errors in the measurement of TCO_2_ may be present, the possibility of error in the input of values into the blood gas apparatus or in reporting acid-base parameters should be evaluated first. The first, and simple, step in identifying the source of large differences between serum TCO_2_ and blood gas HCO_3_^-^ is an independent verification of the calculation of HCO_3_^- ^using a pK’ of 6.1. Equation 3 should be used as the algorithm for this calculation. 
